# A Practical Perspective of the Hematologic Manifestations of Systemic Lupus Erythematosus

**DOI:** 10.7759/cureus.22938

**Published:** 2022-03-07

**Authors:** Juan Camilo Santacruz, Marta Juliana Mantilla, Igor Rueda, Sandra Pulido, Gustavo Rodriguez-Salas, John Londono

**Affiliations:** 1 Spondyloarthropathies Research Group, Universidad de La Sabana, Chía, COL; 2 Rheumatology Department, Universidad Militar Nueva Granada, Bogotá, COL

**Keywords:** autoimmune hemolytic anemia, hematological manifestations, lupus, immune thrombocytopenia, systemic lupus erythematosus

## Abstract

Systemic lupus erythematosus (SLE) is a chronic inflammatory disease with an unknown etiology that can affect any organ or system of the human body. Hematological, renal, or central nervous system manifestations in these patients result in great morbidity because high doses of glucocorticoids, cytotoxic medications, or biological drugs are required to control these manifestations. It is noteworthy that hematological involvement predominates during the first years of the disease and tends to last over time, with the premise that it may be the initial manifestation of the disease. Clear examples of this are the cases of hemolytic anemia and immune thrombocytopenia that can be initially classified as idiopathic or primary to be later classified as secondary when associated with infections, medications, neoplasms, or autoimmune diseases. The spectrum of hematologic manifestations in SLE is very broad, including lymphopenia, anemia, thrombocytopenia, or pancytopenia. In some cases, lymphadenopathy and splenomegaly are also identified. The vast majority of these manifestations denote high disease activity. However, many of these alterations have a multifactorial cause that must be taken into account to adopt a more complete therapeutic approach. The objective of this review is to characterize in detail the hematological manifestations of SLE to offer clinicians a practical vision of its diagnosis and treatment.

## Introduction and background

Hematological abnormalities are common in systemic lupus erythematosus (SLE), both at the time of initial diagnosis and throughout the disease. The most frequent hematologic manifestations include anemia, leukopenia, lymphopenia, thrombocytopenia, lymphadenopathy, and splenomegaly [1]. These abnormalities may be related to the disease itself and/or be caused by immunosuppressive treatment [2]. Alterations in hemostasis have also been widely described, which are caused by the production of autoantibodies that can be prothrombotic (as in the case of antiphospholipid syndrome) or procoagulants by inhibiting the function of coagulation factors favoring hemorrhage [3]. Bone marrow involvement with myelofibrosis changes and aplastic anemia have also been reported [4].

## Review

Redline compromise

Anemia is the most frequent hematological alteration in patients with SLE, occurring in more than 50% of cases [5]. It is important to note that anemia can have a multifactorial cause that requires critical analysis.

Causes of anemia

Anemia of Chronic Disease

Anemia due to chronic disease is the most frequent in patients with SLE, representing approximately one-third of the cases [6]. This results from the upregulation of hepcidin, preventing iron from being incorporated into the red blood cell for development. Hepcidin appears to be regulated by inflammatory cytokines (particularly interleukin (IL) 6), promoting its synthesis by activation of signal transducer and activator of transcription 3 (STAT3) [7]. Other cytokines, including tumor necrosis factor-alpha, interferon-gamma, and IL-1, also interfere with iron homeostasis by reducing cell-surface transferrin receptor concentration and increasing ferritin synthesis [8]. In asymptomatic patients, no treatment other than disease control is usually required; however, some may require treatment with an erythropoiesis-stimulating agent, although to date no controlled clinical trials have been conducted in favor of this intervention [9].

Iron Deficiency

Iron deficiency anemia is also a common cause, usually caused by blood loss (menorrhagia or gastrointestinal bleeding) [9]. Anemia is characteristically normocytic, hypochromic, and aregenerative. Ferritin and/or transferrin saturation levels are low, whereas iron-binding capacity is increased. The cause of bleeding should always be sought, particularly when patients have risk factors such as chronic use of non-steroidal anti-inflammatory drugs (NSAIDs) and oral glucocorticoids. It is important to keep in mind that ferritin is an acute-phase reactant, so it may not accurately reflect iron stores in SLE patients [10]. In general, ferritin values below 100 ng/mL, despite inflammation, reflect significant depletion. Additionally, other tests, such as the soluble transferrin receptor, can be used in case of discordant results [11]. Patients with anemia, both due to iron deficiency and chronic disease, present difficulties for its treatment because chronic inflammation decreases the absorption of oral iron [12]. There are some data on erythropoietic agents and their possible side effects, although if the prescription is considered, hemoglobin levels should be determined after four weeks of treatment and at intervals of approximately four weeks thereafter. Iron should be administered simultaneously with erythropoietic agents because there will be no increase in hemoglobin if iron deficiency is not corrected [12].

Autoimmune Hemolytic Destruction

**Figure 1:**
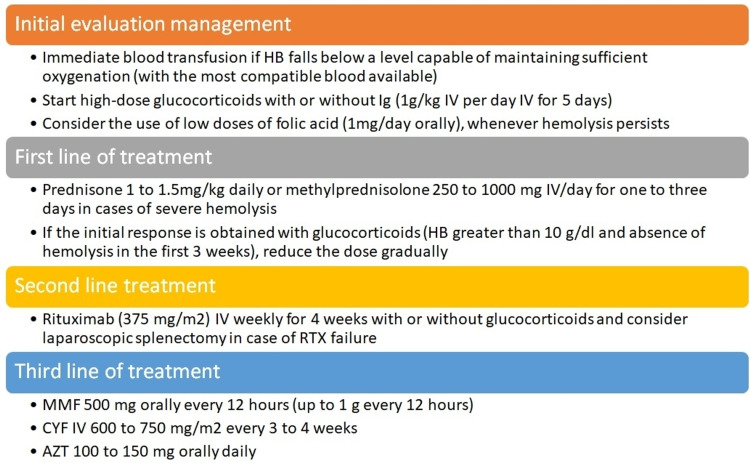
Therapeutic approach for the treatment of autoimmune hemolytic anemia secondary to SLE. AZT: azathioprine; CYF: cyclophosphamide; HB: hemoglobin; MMF: mycophenolate mofetil; Ig: immunoglobulin; IV: intravenous; RTX: rituximab; SLE: systemic lupus erythematosus References: [18-26].

Autoimmune hemolytic anemia (AIHA) occurs in approximately 10% of patients with SLE [13]. Anemia is usually of normal volumes together with predominantly indirect hyperbilirubinemia and increased levels of lactate dehydrogenase (LDH) and reticulocytes. On the other hand, low levels of haptoglobin and spherocytes can be observed in the peripheral blood smear. The combination of increased LDH and reduced haptoglobin is 90% specific for diagnosing hemolysis [14,15]. Autoimmune hemolysis can develop several years before the diagnosis of SLE and can be the main presenting feature [16]. In some cases, AIHA can be accompanied by thrombocytopenia, called Evans syndrome (although its association with SLE is rare) [17]. Neutropenia in the latter syndrome can occur in up to 25% of patients [18]. Warm AIHA is the most common form of presentation, and the evidence for treatment is extrapolated from patients without lupus. The first line of treatment includes high-dose glucocorticoids (prednisone 1 to 1.5 mg/kg daily), evaluating the response with hemoglobin levels that can be reached in up to three weeks. Once hemoglobin greater than 10 g/dL is achieved, the dose should be gradually reduced [19,20]. The general principle of glucocorticoid tapering in AIHA is to use a taper over two to three months [21]. Intravenous methylprednisolone at doses of 250 to 1,000 mg/day for one to three days has been suggested for those with severe and rapidly evolving hemolysis [22]. Only one-third of patients remain in long-term remission once the drug is stopped, and approximately 20-30% require second-line therapies. Other immunosuppressive treatments have also been used, such as rituximab, ofatumumab, mycophenolate, cyclosporine, danazol, intravenous immunoglobulin, or splenectomy [22-24]. Rituximab is becoming the treatment of choice for hemolytic anemia associated with SLE because it allows splenectomy to be avoided together with the risk of complications inherent to surgery and infection by encapsulated germs in the postoperative period [25]. In a meta-analysis of 21 observational studies that included 154 patients with primary or secondary AIHA, the overall response rate for relapsed or refractory disease to rituximab was 79% [26]. It should be taken into account that the therapeutic response may take several weeks, and the relapse rate in one to two years varies from 25% to 50% [27]. It is noteworthy that hemolysis is accompanied by disease activity, in fact, a recently proposed disease activity score (SLE-DAS) has more elements to include less common but serious manifestations, such as myositis, hemolytic anemia, cardiopulmonary manifestations, and gastrointestinal, improving sensitivity compared to SLE disease activity index (SLEDAI), with high specificity and ease of use [28]. Figure 1 describes the initial approach to patients presenting with a hemolytic crisis in the emergency department and the lines of treatment suggested according to current evidence.

Pure Aplastic Anemia

Aplastic anemia is a rare form of bone marrow suppression in which autoantibodies directed against the development of erythrocyte precursors interfere with the genesis of red blood cells [29]. Patients have severe anemia with great hemodynamic repercussions and a low reticulocyte count [30]. In most cases, SLE is diagnosed at the same time as its presentation. The pathogenesis of the disease includes genetic defects that affect the erythropoietic lineage, viral infections (such as parvovirus B19), and factors related to autoimmunity [31,32]. Additionally, there is evidence of anti-erythropoietin antibodies that can neutralize erythropoiesis. It has also been shown that serum from lupus patients can suppress the formation of granulocytic and erythroid colony-stimulating factors in vitro. The immunoglobulin (Ig)G fraction has been isolated from these sera and its action is probably related to the binding of CD34+ hematopoietic progenitor cells but not CD33+ cells [33]. Treatment is based on glucocorticoids together with cyclophosphamide or cyclosporine if an autoimmune etiology is considered [34]. Transfusion support may be required while awaiting the response to drug therapy.

Microangiopathic Hemolytic Anemia

Microangiopathic hemolytic anemia refers to the mechanical narrowing of red blood cells within the circulation, generating schistocytes in the peripheral blood smear. This finding always raises the possibility of thrombotic microangiopathy (TMA) [35]. TMA is seen in 0.5% to 10% of SLE patients, most with poor clinical outcomes [36]. Despite its low incidence, concurrent TMA and SLE is a highly fatal condition with a high mortality rate, ranging from 33.9% to 62.5%, even with the administration of plasmapheresis. Distinguishing between TMA and SLE as the cause of hemolytic anemia is challenging because they share similar clinical features, such as neurologic abnormalities, renal failure, and fever [37]. The clinical benefit of plasmapheresis therapy for TMA syndromes other than thrombotic thrombocytopenic purpura is unclear, and clinical trials have yet to provide recommendations for this therapy. However, plasmapheresis plays a central clinical role in treating TMA syndromes with unclear mechanisms and is a valuable treatment strategy for refractory SLE patients [38]. Previous studies have described that most TMA syndromes developed after the onset of SLE [39,40]. In addition to lupus activity and low ADAMTS13 level, TMA can be caused by coexisting antiphospholipid antibodies, scleroderma, overlapping syndromes, malignant hypertension, infection, and calcineurin inhibitor toxicity [41,42].

Medications

Medications used to control lupus can cause anemia, either via a dose-dependent mechanism or an idiosyncratic reaction. Medications commonly associated with the genesis of anemia are listed in Table 1.

**Table 1:** Medications that can cause anemia in SLE patients. NSAIDs: non-steroidal anti-inflammatory drugs; SLE: systemic lupus erythematosus

Drug	Causal mechanism	References
Cyclophosphamide	Bone marrow suppression	[43]
Hydroxychloroquine	Bone marrow suppression and hemolysis (rare; primarily a theoretical concern in patients with glucose-6-phosphate deficiency)	[44]
Mycophenolate	Bone marrow suppression (reversible)	[45]
Azathioprine	Bone marrow suppression (dose-dependent)	[46]
NSAIDs	Gastrointestinal blood loss with iron deficiency	[47]

Pernicious Anemia

Pernicious anemia, which is caused by the presence of autoantibodies against gastric parietal cells or intrinsic factors, is a very rare cause of anemia in patients with SLE. It can be suspected when high-volume anemia is present together with the presence of hypersegmented neutrophils and other usually mild cytopenias. The prevalence of antibodies against parietal cells appears to be higher in patients with SLE but they are not associated with vitamin B12 deficiency in all cases [48].

White line compromise

Leukopenia

Decreased white blood cell count (leukopenia) usually correlates with disease activity in SLE patients. Counts less than 4000 cells/µL are usually seen in 50% of patients, but only 20% have counts less than 1,000 cells/µL [49]. The pathogenesis of neutropenia in SLE is not fully understood. Proposed potential mechanisms of neutropenia in SLE include increased destruction of peripheral granulocytes, changes in the marginal and splenic pool, and decreased marrow production [50]. Eosinopenia and basophilopenia are rare and of no clinical importance. Lymphopenia is a common finding and predisposes to autoimmunity. The most common type of lymphopenia is T-cell lymphopenia and is highly associated with disease activity, particularly when accompanied by thrombocytopenia [51]. It is important to note that both the American College of Rheumatology and the Systemic Lupus International Collaborating Clinics include leukopenia as a classification criterion. Neutropenia is an uncommon finding; however, it represents an important risk factor for acquiring serious infections [52].

Neutropenia

Neutropenia is characterized by a decrease in the absolute neutrophil count below 1,000 cells/µL. The most frequent causes include viral infections, medications (azathioprine and cyclophosphamide), and hypersplenism; therefore, the treatment of the underlying condition and close monitoring are sufficient in most patients. Neutropenia is only specifically treated with recombinant human granulocyte colony-stimulating factor if it is severe and associated with concomitant infection [53,54]. Functional defects have also been described, involving various autoimmune mechanisms, such as the generation of autoantibodies and/or the action of certain drugs (cyclophosphamide) [55]. Other mechanisms have also been proposed, such as functional alteration due to the deposition of immune complexes, the inhibition of chemotactic factors derived from complement, and/or the action of certain drugs such as glucocorticoids [56].

Lymphopenia

Lymphopenia (defined as an absolute lymphocyte count of <1,500 cells/µL) occurs in 20% to 75% of lupus patients, particularly involving regulatory T-cells [57]. In vitro studies suggest that lymphopenia is caused by the production of autoantibodies against lymphocytes by the genesis of IgG antibodies, correlating inversely with their cell counts and complement levels [58]. Anti-Ro, anti-DNA, and anti-ribonucleoprotein titers have also been reported to be higher in patients with lymphopenia [59]. Autoantibodies against galectin-8 have also been correlated with lymphopenia, particularly in patients with malar rash. However, these autoantibodies have also been described in patients with rheumatoid arthritis and some cases of sepsis [60]. In patients with different subtypes of lupus, T-helper cells decrease more frequently when disease activity is sustained [61]. Lymphopenia does not have a different treatment than the control of lupus. In rare cases of severe lymphopenia, prophylactic antibiotics for *Pneumocystis jirovecii* may be considered [62]. However, the literature is controversial regarding the association between lymphopenia and the risk of severe infections [63]. The SLEDAI score has been positively correlated with lymphopenia and is, together with other hematological changes, a predictor of flare at one year of follow-up [64].

Thrombocytopenia

**Figure 2:**
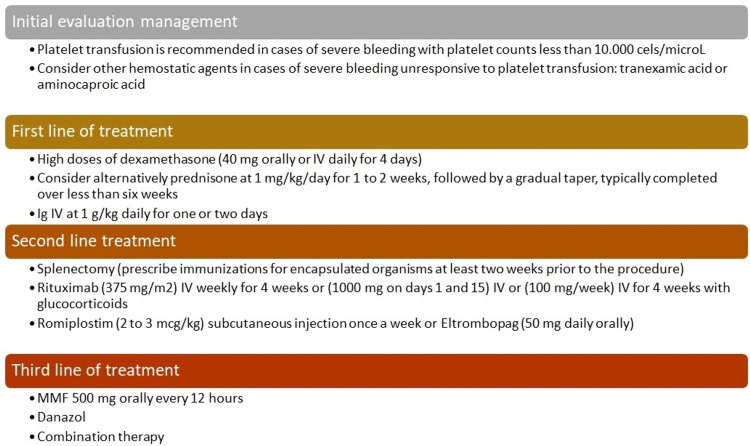
Therapeutic approach of patients with immune thrombocytopenia secondary to SLE with severe bleeding. Ig: immunoglobulin; IV: intravenous; MMF: mycophenolate mofetil; SLE: systemic lupus erythematosus References: [76-84].

Mild thrombocytopenia (platelet count between 100,000 and 150,000 cells/µL) is observed in 25% to 50% of cases, while severe thrombocytopenia (platelets <50,000 cells/µL) is described in only 10% of cases [65]. The most common cause of thrombocytopenia in SLE is immune thrombocytopenia (IT). This entity causes a decrease in the platelet count due to the participation of specific IgG antibodies produced by B-lymphocytes against glycoprotein IIb/IIIa, Ib/IX, and Ia/IIa, although its pathophysiology has not been fully elucidated [66,67]. While the proposed mechanism of thrombocytopenia in IT is the loss of immunological tolerance to certain platelet antigens, thrombocytopenia in SLE can be caused by more complex mechanisms, such as the interaction of antiphospholipid antibodies and antibodies against platelet antigens. It has been shown that the relative risk of thrombocytopenia in patients with antiphospholipid antibodies is greater than four, mainly patients who are positive for lupus anticoagulant [68,69]. IT can originate before the development of lupus, either as a chronic complication or acutely during a flare [70]. Its presentation can precede the onset of lupus for many years, and it has been estimated that between 3% and 15% of isolated IT subsequently debut with the disease [71]. In a case-control study, it was documented that thrombocytopenia was associated with a higher degree of organ damage, being an element highly suggestive of activity [72,73]. Within the initial evaluation of thrombocytopenia in patients with lupus, potentially reversible causes (such as certain medications or infections) should be evaluated. Clinical manifestations include petechiae, purpura, and hemorrhage [74,75]. The potential risk of bleeding depends on the platelet count and the underlying cause of the thrombocytopenia. The probability of bleeding decreases with platelet counts greater than 50,000 cells/µL in the absence of a hemodynamic challenge, such as major trauma or surgery. Platelet transfusion is indicated in cases of hemorrhage with hemodynamic instability, for the prevention of bleeding during an emergency invasive procedure, or when the count is less than 10,000 cells/µL [76]. For IT secondary to SLE, the mainstay of treatment is glucocorticoids. Glucocorticoids are successful in increasing platelet counts in approximately two-thirds of patients, and a platelet response was seen two to five days after treatment. Additionally, remission rates close to 20% have been achieved in monotherapy. Although the mechanism of glucocorticoids is uncertain, it may involve increased apoptosis of autoantibody-producing lymphocytes and decreased activity of macrophages responsible for platelet phagocytosis [77]. The most commonly used treatment regimens are high-dose dexamethasone (40 mg orally or intravenously daily for four days) or alternatively prednisone at a dose of 1 mg/kg daily for one to two weeks. The dexamethasone regimen is preferred as it is faster acting and avoids the toxicity inherent in the long-term use of prednisolone [78-80]. Ig can increase the platelet count between 24 and 48 hours, being more useful before surgery, although its effect is usually transitory. It is also considered for use in patients who cannot tolerate glucocorticoids and are awaiting second-line treatment. IgG is administered intravenously at a dose of 1 g/kg daily for one or two days [81]. It must be taken into account that in 20% to 40% of cases, autoantibodies are directed against the Ibα glycoprotein and can cause thrombocytopenia through a different mechanism (independent of Fc), explaining the failure of Ig administration in some patients (about 25%) [82]. The use of rituximab for the treatment of IT has led to response rates of up to 60%, being an alternative to splenectomy for almost 20 years, with fewer side effects. However, long-term follow-up data showed that only 20-30% of patients maintain remission observing higher response rates in young women before the chronic phase [83]. In a systematic review and meta-analysis, lower doses of rituximab had response rates (overall and complete) similar to the standard dose and could be an alternative therapeutic regimen for IT due to its fewer side effects and lower cost, although controlled clinical studies are required comparing this dose with the standard dose of 375 mg/m^2^ for four weeks [84]. Splenectomy is an alternative therapeutic option for patients with SLE and refractory IT. However, given concerns about the high rate of long-term infections and the advent of rituximab, it has become an obsolete option. If splenectomy is chosen, it is generally preferable to wait at least one year from the time of diagnosis in case a spontaneous remission occurs. Figure 2 presents the therapeutic approach to IT cases who are admitted to the emergency department with severe bleeding.

Pancytopenia

**Figure 3:**
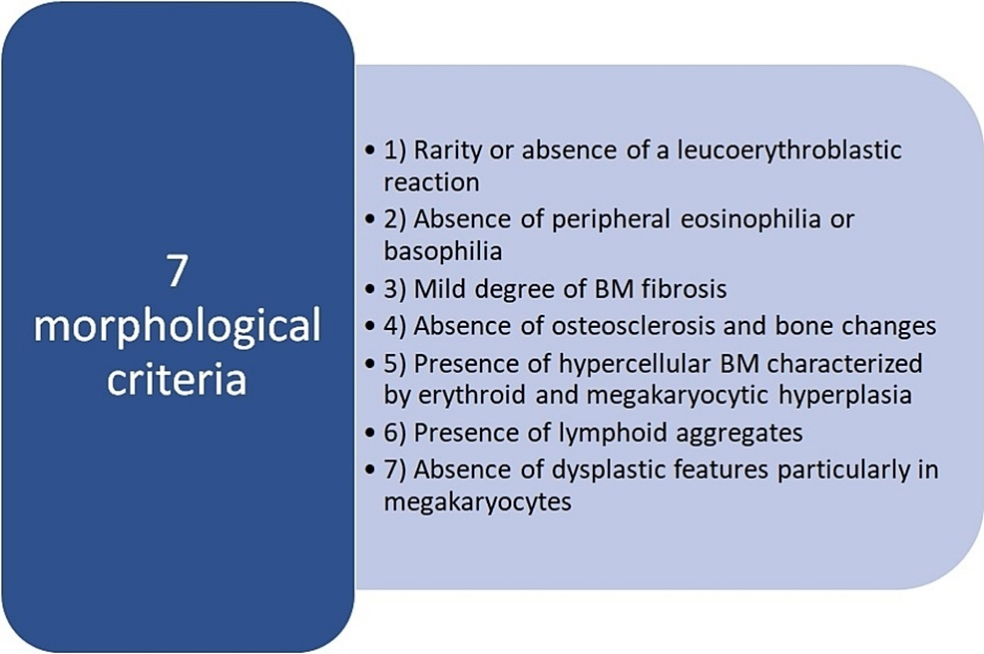
Morphological criteria in BM biopsy for secondary autoimmune myelofibrosis. The diagnosis requires fulfillment of all criteria. BM: bone marrow References: [92,93].

Pancytopenia (defined as a reduction in red blood cell, white blood cell, and platelet counts) is less common than isolated cytopenias but can occur in SLE. The most frequent findings in bone marrow aspirate are hypocellularity and bone marrow necrosis attributed to autoimmune mechanisms of the disease [85,86]. There are various causes, some of which are much more deadly, such as macrophage activation syndrome, which occurs due to a loss of immune regulation that leads to the massive activation of macrophages in the bone marrow and other tissues. The clinical manifestations include fever, hepatosplenomegaly, lymphadenopathy, neurological symptoms, skin rash, cytopenias, very high serum ferritin levels, and liver function abnormalities. Hemophagocytosis is often present in the bone marrow but is neither pathognomonic nor sufficient to establish the diagnosis on its own [87]. Cytotoxic or myelosuppressive drugs are always a cause of pancytopenia to consider. Cell count recovery usually occurs within a few days to weeks once the medication is stopped. Mechanisms of drug-associated cytopenias include allergic reactions that affect bone marrow production or increase peripheral destruction. Allergic reactions can be influenced by the patient’s immunological background (such as human leukocyte antigen type), associated comorbidities, and pharmacogenomic makeup [88]. Myelofibrosis is another cause of pancytopenia to consider and is characterized by the clonal appearance of myeloid stem cells accompanied by stromal production of fibrinous ground substance. These changes can be attributed to primary myeloproliferative disorders or can be a manifestation of various malignant, endocrine, or inflammatory diseases [89]. However, myelofibrosis has not been included in the classification criteria for SLE, despite the fact that there is ample evidence that it is a definitive finding, although its presentation is rare. The diagnosis of autoimmune myelofibrosis is based on the morphological characteristics detected in the bone marrow. Currently, the gold standard remains biopsy, which is an invasive diagnostic method. Recently, several biomarkers have been introduced for primary myelofibrosis, such as circulating YKL-40 and GATA-1, but not for SLE-associated myelofibrosis [90,91]. Vergara-Lluri et al. proposed the morphological criteria in bone marrow biopsy for secondary autoimmune myelofibrosis based on their study population, where 69% had an autoimmune disease (Figure 3) [92,93]. The main treatment for myelofibrosis associated with SLE is glucocorticoids.

Other relevant hematological alterations

Lymphadenopathy

Lymphadenopathy (enlargement of one or more lymph nodes) occurs in approximately 50% of lupus patients. They are usually soft, painless, of variable size, and located mainly in the cervical, axillary, and inguinal regions. They are usually seen early in the disease or in active cases, rapidly diminishing in size with glucocorticoid initiation. Biopsies generally reveal areas of follicular hyperplasia and necrosis; the appearance of hematoxylin bodies is highly suggestive of SLE, although this finding is unusual [94]. Enlarged lymph nodes in SLE patients may also be due to infection (e.g., mononucleosis) or lymphoproliferative diseases, such as lymphoma or chronic lymphocytic leukemia. Several cohort series and case-control studies have shown that the risks of non-Hodgkin’s lymphoma are approximately three to four times higher in SLE compared to the general population. The risk of Hodgkin’s lymphoma may also be increased [95].

Leukocytosis

Leukocytosis and neutrophilia can occur from various causes in SLE, either from infection, from the use of high doses of glucocorticoids, or, less frequently, from disease activity. Neutrophilia with a left shift always suggests infection [96].

Thrombocytosis

Thrombocytosis (increased platelet count of ≥450,000 cells/µL) can be presented in patients with SLE in cases of infection, iron deficiency, or severe hemorrhage, although it is less common than thrombocytopenia. Another cause to consider is hyposplenism or autosplenectomy, which may be related to antiphospholipid antibodies or antiphospholipid syndrome [97,98]. No treatment is required for reactive thrombocytosis due to the loss of splenic function. However, if the thrombocytosis is related to a myeloproliferative neoplasm, therapy to reduce the platelet count and/or prevent thromboembolic complications may be necessary.

Splenomegaly

Splenomegaly occurs in 10% to 46% of SLE patients, particularly during active disease. The mechanism is not clearly understood. However, splenomegaly is not associated with cytopenias, although mild cytopenias can often be seen due to the accumulation of blood and blood cells in the spleen [99,100].

Emerging therapies

Combination Therapy (Rituximab-Belimumab)

The B-cell activating factor may be involved in the failure of B-cell depleting therapy with rituximab in IT by promoting the emergence of splenic long-lived plasma cells. Based on this demonstrated hypothesis in murine models, a phase IIb study was conducted (RITUX-PLUS) investigating the safety and efficacy of rituximab at a fixed dose of 1,000 mg, two weeks apart, combined with five intravenous infusions of belimumab (10 mg/kg) at weeks zero, two, four, eight, and twelve for adults with primary persistent or chronic IT. The primary endpoint was the total number of patients achieving an overall response (complete response (CR) and response (R) at week 52). CR was defined by platelet counts of >100×10^9^/L and R by platelet count 30-100×10^9^/L with at least a two-fold increase from baseline according to international definitions. Combination therapy led to an overall response rate of 80% with a 66.7% CR rate at one year. The main limitations of this single-center, exploratory pilot trial were the sample size and open design. No serious adverse events, infections, or severe hypogammaglobulinemia were documented [101]. The study did not include patients with SLE, although it is not ruled out that combined therapy may have similar efficacy as observed in other clinical scenarios such as refractory lupus nephritis. More clinical studies are required to corroborate this hypothesis.

Daratumumab

Daratumumab is a human monoclonal anti-CD38, plasma cell-depleting drug approved for the treatment of multiple myeloma. In SLE, long-lived plasma cells are pathogenic and considered a therapeutic challenge because these cells secrete autoantibodies while being treated with immunosuppressive and B-cell-targeted therapies. Because long-lived plasma cells express CD38, the therapeutic potential of daratumumab in SLE was studied in one patient who had Evans syndrome and vasculitic skin lesions. The patient was treated with subcutaneous daratumumab (16 mg per kg, once a week for four weeks) in addition to continuous immunosuppression, and was followed four months later with belimumab as maintenance therapy. Subsequently, the hemolytic anemia and skin lesions resolved and her platelet counts reached normal levels [102]. It is likely that daratumumab could become a promising therapy in cases of AIHA secondary to lupus.

## Conclusions

Hematological abnormalities are common in patients with SLE. Although these may be secondary to the disease, concomitant conditions or side effects of immunosuppressive drugs must be ruled out. The study of these alterations should be aimed at identifying their main etiology and evaluating other causes that may be potentially reversible. Clinicians must keep in mind that many of the hematological alterations have a multifactorial cause, sometimes requiring combined therapeutic interventions. Bone marrow biopsy should be considered in cases of pancytopenia or anemia of unknown cause to rule out less common hematologic disorders, such as aplastic anemia and autoimmune myelofibrosis. More clinical studies on emerging therapies that include patients with SLE are required to validate their clinical utility in refractory cases.

## References

[1] Aleem A, Al Arfaj AS, khalil N, Alarfaj H (2014). Haematological abnormalities in systemic lupus erythematosus. Acta Reumatol Port.

[2] Velo-García A, Castro SG, Isenberg DA (2016). The diagnosis and management of the haematologic manifestations of lupus. J Autoimmun.

[3] Giannouli S, Voulgarelis M, Ziakas PD, Tzioufas AG (2006). Anaemia in systemic lupus erythematosus: from pathophysiology to clinical assessment. Ann Rheum Dis.

[4] Habib GS, Saliba WR, Froom P (2002). Pure red cell aplasia and lupus. Semin Arthritis Rheum.

[5] Fayyaz A, Igoe A, Kurien BT, Danda D, James JA, Stafford HA, Scofield RH (2015). Haematological manifestations of lupus. Lupus Sci Med.

[6] Voulgarelis M, Kokori SI, Ioannidis JP, Tzioufas AG, Kyriaki D, Moutsopoulos HM (2000). Anaemia in systemic lupus erythematosus: aetiological profile and the role of erythropoietin. Ann Rheum Dis.

[7] Verga Falzacappa MV, Vujic Spasic M, Kessler R, Stolte J, Hentze MW, Muckenthaler MU (2007). STAT3 mediates hepatic hepcidin expression and its inflammatory stimulation. Blood.

[8] Baker JF, Ghio AJ (2009). Iron homoeostasis in rheumatic disease. Rheumatology (Oxford).

[9] Levine AB, Erkan D (2011). Clinical assessment and management of cytopenias in lupus patients. Curr Rheumatol Rep.

[10] Newman K, Owlia MB, El-Hemaidi I, Akhtari M (2013). Management of immune cytopenias in patients with systemic lupus erythematosus - old and new. Autoimmun Rev.

[11] Mittal S, Agarwal P, Wakhlu A, Kumar A, Mehrotra R, Mittal S (2016). Anaemia in systemic lupus erythematosus based on iron studies and soluble transferrin receptor levels. J Clin Diagn Res.

[12] Weiss G, Goodnough LT (2005). Anemia of chronic disease. N Engl J Med.

[13] Jeffries M, Hamadeh F, Aberle T (2008). Haemolytic anaemia in a multi-ethnic cohort of lupus patients: a clinical and serological perspective. Lupus.

[14] Marchand A, Galen RS, Van Lente F (1980). The predictive value of serum haptoglobin in hemolytic disease. JAMA.

[15] Barcellini W, Fattizzo B, Zaninoni A (2014). Clinical heterogeneity and predictors of outcome in primary autoimmune hemolytic anemia: a GIMEMA study of 308 patients. Blood.

[16] Kokori SI, Ioannidis JP, Voulgarelis M, Tzioufas AG, Moutsopoulos HM (2000). Autoimmune hemolytic anemia in patients with systemic lupus erythematosus. Am J Med.

[17] Michel M, Chanet V, Dechartres A (2009). The spectrum of Evans syndrome in adults: new insight into the disease based on the analysis of 68 cases. Blood.

[18] Costallat GL, Appenzeller S, Costallat LT (2012). Evans syndrome and systemic lupus erythematosus: clinical presentation and outcome. Joint Bone Spine.

[19] Gomard-Mennesson E, Ruivard M, Koenig M (2006). Treatment of isolated severe immune hemolytic anaemia associated with systemic lupus erythematosus: 26 cases. Lupus.

[20] Karpouzas AG (2019). Hematologic and lymphoid abnormalities in SLE. Dubois’ Lupus Erythematosus and Related Syndromes.

[21] Zanella A, Barcellini W (2014). Treatment of autoimmune hemolytic anemias. Haematologica.

[22] Go RS, Winters JL, Kay NE (2017). How I treat autoimmune hemolytic anemia. Blood.

[23] Poulet A, Jarrot PA, Mazodier K, Jean R, Kaplanski G (2019). Successful treatment of systemic lupus erythematosus-related refractory autoimmune hemolytic anemia with ofatumumab. Lupus.

[24] Akpek G, McAneny D, Weintraub L (1999). Comparative response to splenectomy in Coombs-positive autoimmune hemolytic anemia with or without associated disease. Am J Hematol.

[25] Dierickx D, Kentos A, Delannoy A (2015). The role of rituximab in adults with warm antibody autoimmune hemolytic anemia. Blood.

[26] Reynaud Q, Durieu I, Dutertre M (2015). Efficacy and safety of rituximab in auto-immune hemolytic anemia: a meta-analysis of 21 studies. Autoimmun Rev.

[27] Maung SW, Leahy M, O'Leary HM (2013). A multi-centre retrospective study of rituximab use in the treatment of relapsed or resistant warm autoimmune haemolytic anaemia. Br J Haematol.

[28] Jesus D, Matos A, Henriques C (2019). Derivation and validation of the SLE Disease Activity Score (SLE-DAS): a new SLE continuous measure with high sensitivity for changes in disease activity. Ann Rheum Dis.

[29] Hara A, Wada T, Kitajima S (2008). Combined pure red cell aplasia and autoimmune hemolytic anemia in systemic lupus erythematosus with anti-erythropoietin autoantibodies. Am J Hematol.

[30] Fisch P, Handgretinger R, Schaefer HE (2000). Pure red cell aplasia. Br J Haematol.

[31] Young NS, Brown KE (2004). Parvovirus B19. N Engl J Med.

[32] Young NS, Maciejewski J (1997). The pathophysiology of acquired aplastic anemia. N Engl J Med.

[33] Liu H, Ozaki K, Matsuzaki Y, Abe M, Kosaka M, Saito S (1995). Suppression of haematopoiesis by IgG autoantibodies from patients with systemic lupus erythematosus (SLE). Clin Exp Immunol.

[34] Winkler A, Jackson RW, Kay DS, Mitchell E, Carmignani S, Sharp GC (1988). High-dose intravenous cyclophosphamide treatment of systemic lupus erythematosus-associated aplastic anemia. Arthritis Rheum.

[35] George JN, Nester CM (2014). Syndromes of thrombotic microangiopathy. N Engl J Med.

[36] Pattanashetti N, Anakutti H, Ramachandran R, Rathi M, Sharma A, Nada R, Gupta KL (2017). Effect of thrombotic microangiopathy on clinical outcomes in Indian patients with lupus nephritis. Kidney Int Rep.

[37] Kwok SK, Ju JH, Cho CS, Kim HY, Park SH (2009). Thrombotic thrombocytopenic purpura in systemic lupus erythematosus: risk factors and clinical outcome: a single centre study. Lupus.

[38] Kronbichler A, Brezina B, Quintana LF, Jayne DR (2016). Efficacy of plasma exchange and immunoadsorption in systemic lupus erythematosus and antiphospholipid syndrome: a systematic review. Autoimmun Rev.

[39] Letchumanan P, Ng HJ, Lee LH, Thumboo J (2009). A comparison of thrombotic thrombocytopenic purpura in an inception cohort of patients with and without systemic lupus erythematosus. Rheumatology (Oxford).

[40] Chen MH, Chen MH, Chen WS, Mu-Hsin Chang P, Lee HT, Lin HY, Huang DF (2011). Thrombotic microangiopathy in systemic lupus erythematosus: a cohort study in North Taiwan. Rheumatology (Oxford).

[41] Yue C, Su J, Gao R (2018). Characteristics and outcomes of patients with systemic lupus erythematosus-associated thrombotic microangiopathy, and their acquired ADAMTS13 inhibitor profiles. J Rheumatol.

[42] Song D, Wu LH, Wang FM (2013). The spectrum of renal thrombotic microangiopathy in lupus nephritis. Arthritis Res Ther.

[43] Hall AG, Tilby MJ (1992). Mechanisms of action of, and modes of resistance to, alkylating agents used in the treatment of haematological malignancies. Blood Rev.

[44] Kuraitis D, Murina A (2020). Facts, not fear: safety of hydroxychloroquine. Am J Med Sci.

[45] Kiang TK, Ensom MH (2019). Exposure-toxicity relationships of mycophenolic acid in adult kidney transplant patients. Clin Pharmacokinet.

[46] Formea CM, Myers-Huentelman H, Wu R (2004). Thiopurine S-methyltransferase genotype predicts azathioprine-induced myelotoxicity in kidney transplant recipients. Am J Transplant.

[47] Upadhyay R, Torley HI, McKinlay AW, Sturrock RD, Russell RI (1990). Iron deficiency anaemia in patients with rheumatic disease receiving non-steroidal anti-inflammatory drugs: the role of upper gastrointestinal lesions. Ann Rheum Dis.

[48] Picceli VF, Skare TL, Nisihara R, Kotze L, Messias-Reason I, Utiyama SR (2013). Spectrum of autoantibodies for gastrointestinal autoimmune diseases in systemic lupus erythematosus patients. Lupus.

[49] Harvey AM, Shulman LE, Tumulty PA, Conley CL, Schoenrich EH (1954). Systemic lupus erythematosus: review of the literature and clinical analysis of 138 cases. Medicine (Baltimore).

[50] Starkebaum G, Price TH, Lee MY, Arend WP (1978). Autoimmune neutropenia in systemic lupus erythematosus. Arthritis Rheum.

[51] Mirzayan MJ, Schmidt RE, Witte T (2000). Prognostic parameters for flare in systemic lupus erythematosus. Rheumatology (Oxford).

[52] Martínez-Baños D, Crispín JC, Lazo-Langner A, Sánchez-Guerrero J (2006). Moderate and severe neutropenia in patients with systemic lupus erythematosus. Rheumatology (Oxford).

[53] Budman DR, Steinberg AD (1977). Hematologic aspects of systemic lupus erythematosus. Current concepts. Ann Intern Med.

[54] Kondo H, Date Y, Sakai Y, Akimoto M (1994). Effective simultaneous rhG-CSF and methylprednisolone "pulse" therapy in agranulocytosis associated with systemic lupus erythematosus. Am J Hematol.

[55] Smith CK, Kaplan MJ (2015). The role of neutrophils in the pathogenesis of systemic lupus erythematosus. Curr Opin Rheumatol.

[56] Hadley AG, Byron MA, Chapel HM, Bunch C, Holburn AM (1987). Anti-granulocyte opsonic activity in sera from patients with systemic lupus erythematosus. Br J Haematol.

[57] Rivero SJ, Díaz-Jouanen E, Alarcón-Segovia D (1978). Lymphopenia in systemic lupus erythematosus. Clinical, diagnostic, and prognostic significance. Arthritis Rheum.

[58] Noguchi M, Iwamori M, Hirano T, Kobayashi S, Hashimoto H, Hirose S, Nagai Y (1992). Autoantibodies to T and B cell lines detected in serum samples from patients with systemic lupus erythematosus with lymphopenia and hypocomplementaemia. Ann Rheum Dis.

[59] García-Valladares I, Atisha-Fregoso Y, Richaud-Patin Y (2006). Diminished expression of complement regulatory proteins (CD55 and CD59) in lymphocytes from systemic lupus erythematosus patients with lymphopenia. Lupus.

[60] Massardo L, Metz C, Pardo E (2009). Autoantibodies against galectin-8: their specificity, association with lymphopenia in systemic lupus erythematosus and detection in rheumatoid arthritis and acute inflammation. Lupus.

[61] Wenzel J, Bauer R, Bieber T, Boehm I (2000). Presence of antinuclear antibodies in patients with lupus erythematosus is correlated with diminished T-helper cells. Br J Dermatol.

[62] Vananuvat P, Suwannalai P, Sungkanuparph S, Limsuwan T, Ngamjanyaporn P, Janwityanujit S (2011). Primary prophylaxis for Pneumocystis jirovecii pneumonia in patients with connective tissue diseases. Semin Arthritis Rheum.

[63] Carli L, Tani C, Vagnani S, Signorini V, Mosca M (2015). Leukopenia, lymphopenia, and neutropenia in systemic lupus erythematosus: prevalence and clinical impact--a systematic literature review. Semin Arthritis Rheum.

[64] Li C, Mu R, Lu XY, He J, Jia RL, Li ZG (2014). Antilymphocyte antibodies in systemic lupus erythematosus: association with disease activity and lymphopenia. J Immunol Res.

[65] Keeling DM, Isenberg DA (1993). Haematological manifestations of systemic lupus erythematosus. Blood Rev.

[66] Michel M, Lee K, Piette JC, Fromont P, Schaeffer A, Bierling P, Godeau B (2002). Platelet autoantibodies and lupus-associated thrombocytopenia. Br J Haematol.

[67] Ahmed AE, Peter JB, Shoenfeld YY (1998). ANCA testing. New developments and clinical implications. Clin Rev Allergy Immunol.

[68] Abu-Shakra M, Gladman DD, Urowitz MB, Farewell V (1995). Anticardiolipin antibodies in systemic lupus erythematosus: clinical and laboratory correlations. Am J Med.

[69] Morgan M, Downs K, Chesterman CN, Biggs JC (1993). Clinical analysis of 125 patients with the lupus anticoagulant. Aust N Z J Med.

[70] Ziakas PD, Giannouli S, Zintzaras E, Tzioufas AG, Voulgarelis M (2005). Lupus thrombocytopenia: clinical implications and prognostic significance. Ann Rheum Dis.

[71] Karpatkin S (1980). Autoimmune thrombocytopenic purpura. Blood.

[72] Mok CC, Lee KW, Ho CT, Lau CS, Wong RW (2000). A prospective study of survival and prognostic indicators of systemic lupus erythematosus in a southern Chinese population. Rheumatology (Oxford).

[73] Miller MH, Urowitz MB, Gladman DD (1983). The significance of thrombocytopenia in systemic lupus erythematosus. Arthritis Rheum.

[74] Mithoowani S, Cervi A, Shah N (2020). Management of major bleeds in patients with immune thrombocytopenia. J Thromb Haemost.

[75] Neunert C, Noroozi N, Norman G (2015). Severe bleeding events in adults and children with primary immune thrombocytopenia: a systematic review. J Thromb Haemost.

[76] Neunert C, Lim W, Crowther M, Cohen A, Solberg L Jr, Crowther MA (2011). The American Society of Hematology 2011 evidence-based practice guideline for immune thrombocytopenia. Blood.

[77] Mizutani H, Furubayashi T, Imai Y (1992). Mechanisms of corticosteroid action in immune thrombocytopenic purpura (ITP): experimental studies using ITP-prone mice, (NZW x BXSB) F1. Blood.

[78] (2020). Neunert C, Terrell DR, Arnold DM, et al. American Society of Hematology 2019 guidelines for immune thrombocytopenia. Blood Adv. 2019;3(23):3829-3866. Blood Adv.

[79] Provan D, Arnold DM, Bussel JB (2019). Updated international consensus report on the investigation and management of primary immune thrombocytopenia. Blood Adv.

[80] Mithoowani S, Gregory-Miller K, Goy J (2016). High-dose dexamethasone compared with prednisone for previously untreated primary immune thrombocytopenia: a systematic review and meta-analysis. Lancet Haematol.

[81] Ammann EM, Haskins CB, Fillman KM (2016). Intravenous immune globulin and thromboembolic adverse events: a systematic review and meta-analysis of RCTs. Am J Hematol.

[82] Webster ML, Sayeh E, Crow M, Chen P, Nieswandt B, Freedman J, Ni H (2006). Relative efficacy of intravenous immunoglobulin G in ameliorating thrombocytopenia induced by antiplatelet GPIIbIIIa versus GPIbalpha antibodies. Blood.

[83] Lucchini E, Zaja F, Bussel J (2019). Rituximab in the treatment of immune thrombocytopenia: what is the role of this agent in 2019?. Haematologica.

[84] Li Y, Shi Y, He Z, Chen Q, Liu Z, Yu L, Wang C (2019). The efficacy and safety of low-dose rituximab in immune thrombocytopenia: a systematic review and meta-analysis. Platelets.

[85] Voulgarelis M, Giannouli S, Tasidou A, Anagnostou D, Ziakas PD, Tzioufas AG (2006). Bone marrow histological findings in systemic lupus erythematosus with hematologic abnormalities: a clinicopathological study. Am J Hematol.

[86] Wanitpongpun C, Teawtrakul N, Mahakkanukrauh A, Siritunyaporn S, Sirijerachai C, Chansung K (2012). Bone marrow abnormalities in systemic lupus erythematosus with peripheral cytopenia. Clin Exp Rheumatol.

[87] Lambotte O, Khellaf M, Harmouche H (2006). Characteristics and long-term outcome of 15 episodes of systemic lupus erythematosus-associated hemophagocytic syndrome. Medicine (Baltimore).

[88] Sutton JF, Stacey M, Kearns WG, Rieg TS, Young NS, Liu JM (2004). Increased risk for aplastic anemia and myelodysplastic syndrome in individuals lacking glutathione S-transferase genes. Pediatr Blood Cancer.

[89] Aziz AR, Mohammadian Y, Ruby C, Momin Z, Kumar A, Griciene P, Gintautas J (2004). Systemic lupus erythematosus presenting with pancytopenia due to bone marrow myelofibrosis in a 22-year-old male. Clin Adv Hematol Oncol.

[90] Bjørn ME, Andersen CL, Jensen MK, Hasselbalch HC (2014). Circulating YKL-40 in myelofibrosis a potential novel biomarker of disease activity and the inflammatory state. Eur J Haematol.

[91] Lally J, Boasman K, Brown L (2019). GATA-1: a potential novel biomarker for the differentiation of essential thrombocythemia and myelofibrosis. J Thromb Haemost.

[92] Vergara-Lluri ME, Piatek CI, Pullarkat V, Siddiqi IN, O'Connell C, Feinstein DI, Brynes RK (2014). Autoimmune myelofibrosis: an update on morphologic features in 29 cases and review of the literature. Hum Pathol.

[93] Marcellino B, El Jamal SM, Mascarenhas JO (2018). Distinguishing autoimmune myelofibrosis from primary myelofibrosis. Clin Adv Hematol Oncol.

[94] Kojima M, Motoori T, Asano S, Nakamura S (2007). Histological diversity of reactive and atypical proliferative lymph node lesions in systemic lupus erythematosus patients. Pathol Res Pract.

[95] Cao L, Tong H, Xu G (2015). Systemic lupus erythematous and malignancy risk: a meta-analysis. PLoS One.

[96] Boumpas DT, Chrousos GP, Wilder RL, Cupps TR, Balow JE (1993). Glucocorticoid therapy for immune-mediated diseases: basic and clinical correlates. Ann Intern Med.

[97] Abid N (2013). Thrombocytosis in a patient with systemic lupus. J Pak Med Assoc.

[98] Childs JC, Adelizzi RA, Dabrow MB, Freed N (1994). Splenic hypofunction in systemic lupus erythematosus. J Am Osteopath Assoc.

[99] Piliero P, Furie R (1990). Functional asplenia in systemic lupus erythematosus. Semin Arthritis Rheum.

[100] Thipphavong S, Duigenan S, Schindera ST, Gee MS, Philips S (2014). Nonneoplastic, benign, and malignant splenic diseases: cross-sectional imaging findings and rare disease entities. AJR Am J Roentgenol.

[101] Mahévas M, Azzaoui I, Crickx E (2021). Efficacy, safety and immunological profile of combining rituximab with belimumab for adults with persistent or chronic immune thrombocytopenia: results from a prospective phase 2b trial. Haematologica.

[102] Ostendorf L, Burns M, Durek P (2020). Targeting CD38 with daratumumab in refractory systemic lupus erythematosus. N Engl J Med.

